# Left Ventricular Mass Regression following Implantation of St. Jude Medical Trifecta Aortic Bioprosthesis

**DOI:** 10.1186/1749-8090-10-S1-A308

**Published:** 2015-12-16

**Authors:** JA Chacko, J Edlin, AH Sepehripour, SG Ambekar, KS Lall

**Affiliations:** 1 St.Bartholomew's Hospital, London, UK

## Background/Introduction

The St. Jude Medical Trifecta aortic supra-annular bioprosthesis is regarded as the next generation in pericardial stented tissue valves. The unique design of tissue leaflets attached to the exterior of the valve stent provides unrivalled in-vivo mean gradients and haemodynamics.

## Aims/Objectives

The aim of this prospective study was to evaluate midterm left ventricular (LV) mass regression following implantation for aortic stenosis.

## Method

One hundred and seventy two consecutive patients undergoing aortic valve replacement using the St. Jude Medical Trifecta valve at a single UK centre over a 48-month period were included in this study. Patients undergoing concomitant cardiac procedures were included. All implanted valves were 19, 21, 23, 25, 27 & 29 mm in size. Patients underwent both pre-operative and post-operative transthoracic echocardiography. Two-dimensional measurements of the left ventricle were used to calculate LV mass using the Devereux equation.

## Results

30 patients had the adequate 2-dimensional left ventricular measurements recorded to calculate both pre- and postoperative left ventricular mass. Valve Sizes were 21 mm (n = 7), 23 mm (n = 15), 25 mm (n = 6) and 27 mm (n = 2). Overall absolute left ventricular mass regression was 18.1% ± 23.8%. Mean preoperative LV mass was 247.8g ± 102.5 g and mean postoperative LV mass was 200.7 ± 74.1g. Regression of LV Mass Index was -30.66 g/m2.

## Discussion/Conclusion

Utilising available 2-D Measurements in this group we observe regression of LV mass & LV Mass Index post aortic valve replacement with the Trifecta bioprosthetic valve. However, further consistent 2-D measurements are required across the cohort to establish this relationship.

